# Posterior Fossa Meningioma with Endolymphatic Sac Compression: Resolution of Dizziness after Surgery

**DOI:** 10.1055/a-2882-7511

**Published:** 2026-06-09

**Authors:** Cody John Schroeder, Daniel Mitchell, Luke Silveira, Brandon Liebelt, George Kurien

**Affiliations:** 1 Division of Otolaryngology – Head and Neck SurgeryDepartment of Surgery 2092 University of VermontBurlingtonVermontUnited States; 2 Larner College of Medicine 2092 University of VermontBurlingtonVermontUnited States; 3 Division of NeurosurgeryDepartment of Surgery 2090 University of Vermont Medical CenterBurlingtonVermontUnited States

**Keywords:** posterior fossa meningioma, dizziness, endolymphatic sac compression, vHIT

## Abstract

Dizziness is a prevalent clinical complaint, and determining the underlying etiology can be challenging. Posterior fossa masses have been observed to cause vertigo, with presentations very similar to Meniere’s disease. In this report, we describe a case of a patient who presented with recurrent episodes of Meniere’s-like vertigo in the setting of a posterior fossa meningioma, who underwent surgical resection of the tumor with significant improvement postoperatively. We postulate that these symptoms were caused by secondary endolymphatic hydrops and were able to demonstrate improvement in high-frequency vestibulo-ocular reflex with pre- and postoperative video head impulse testing.

## Introduction

Dizziness is a prevalent clinical complaint, accounting for up to 5% of all primary care visits, with up to 30% of patients experiencing it as a secondary complaint. 1 Diagnosing the etiology of dizziness is challenging due to the vast potential differential and the difficulty patients face in describing their symptoms. Dizziness is characteristically divided into four categories: true vertigo, presyncope, disequilibrium, and psychological dizziness. 2 True vertigo, the illusory sensation of motion, accounts for approximately 55% of cases, often accompanied by nausea, vomiting, and nystagmus. 3

True vertigo is further classified into neurologic (central) or vestibular (peripheral), the former due to insults to neuronal pathways, disrupting vestibular transduction, while the latter stems from direct insult to the vestibular labyrinth. 4 Among peripheral causes, benign paroxysmal positional vertigo, vestibular neuronitis, and Meniere’s disease account for approximately 90% of all cases of peripheral vertigo. 2

Posterior fossa masses have been observed to cause vertiginous presentations; however, the estimated prevalence is <1%. 5 Of these cases, petrous temporal bone meningiomas involving the endolymphatic sac are even less common, representing approximately 29% of the cases revealed in our search. In this case report, we present a 74-year-old female patient with a posterior fossa mass initially presenting as recurrent episodes of Meniere’s-like vertigo who underwent surgical resection of the tumor with significant improvement postoperatively.

## Case Report

**Fig. 1:**
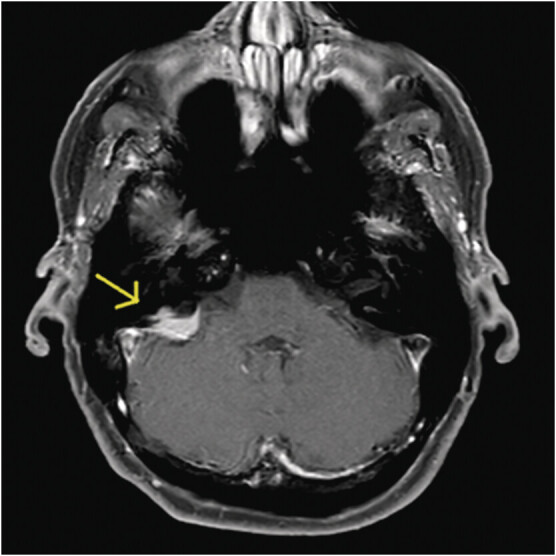
T1 postcontrast MRI image showing meningioma with invasion of the vestibular aqueduct.

**Fig. 2:**
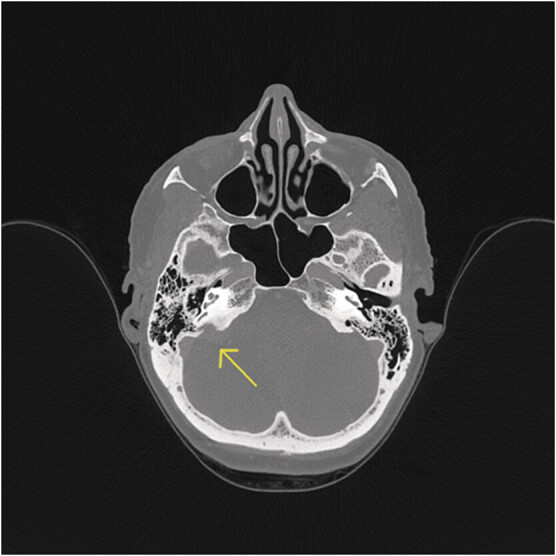
CT scan showing bony changes in the vestibular aqueduct.

A 73-year-old woman with a history of migraines presented with dizziness and ataxia in the setting of a right posterior fossa meningioma. She reported approximately 15 years of intermittent symptoms with episodes of vertigo one to three times per week. Episodes lasted in the range of hours and were associated with nausea and vomiting. She was mostly asymptomatic between episodes. She also reported a gradual decline in hearing on the right in the past 5 years with associated tinnitus. MRI showed a right posterior fossa lesion with imaging findings most consistent with a meningioma based along the petrous temporal bone ( Fig. 1 ). There appeared to be invasion of the bony operculum of the vestibular aqueduct, with confirmatory findings on CT scan ( Fig. 2 ).

**Fig. 3:**
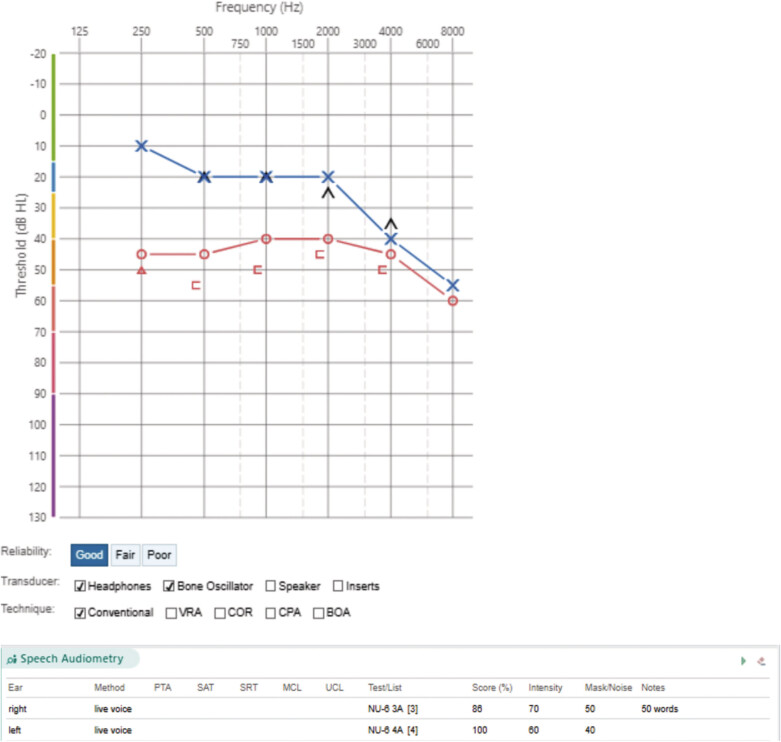
Preoperative pure tone audiogram showing a moderate right sensorineural hearing loss.

**Fig. 4:**
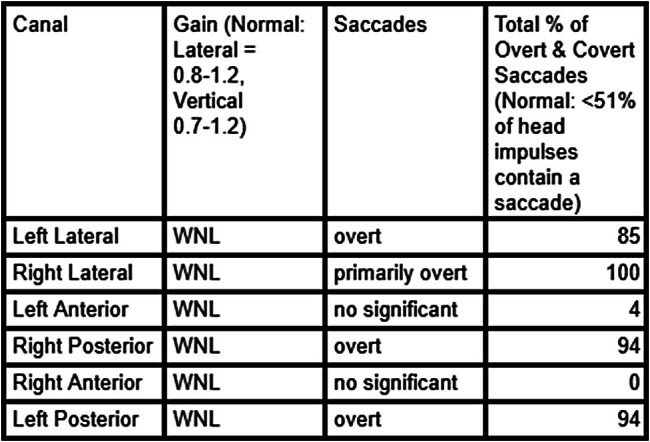
Preoperative video head impulse testing (vHIT) showing normal gain with overt saccades on the right. Noted also are overt saccades in the left lateral and posterior semicircular canals.

She underwent further investigation with a pure tone audiogram ( Fig. 3 ), video head impulse testing (vHIT; Fig. 4 ), and videonystagmography. In summary, she was found to have an asymmetric right-sided moderate sensorineural hearing loss with slightly reduced speech discrimination at 86%, right-sided high-frequency vestibulo-ocular reflex (VOR) deficit on vHIT, and 71% caloric weakness on the right.

The patient underwent resection of the tumor via a right retrosigmoid approach. Intraoperatively, the tumor was found to be involving the temporal bone in the region of the endolymphatic sac and duct without obvious invasion. Gross total resection (Simpson Grade I) was performed, bony areas of erosion were drilled, and the exposed air cells were waxed and covered with pericranium. Frozen and permanent pathology confirmed the diagnosis of meningioma. Final permanent pathology showed CNS WHO Grade 1 with a K _i_ -67 index of 2.8% and scattered positive CD3.

**Fig. 5:**
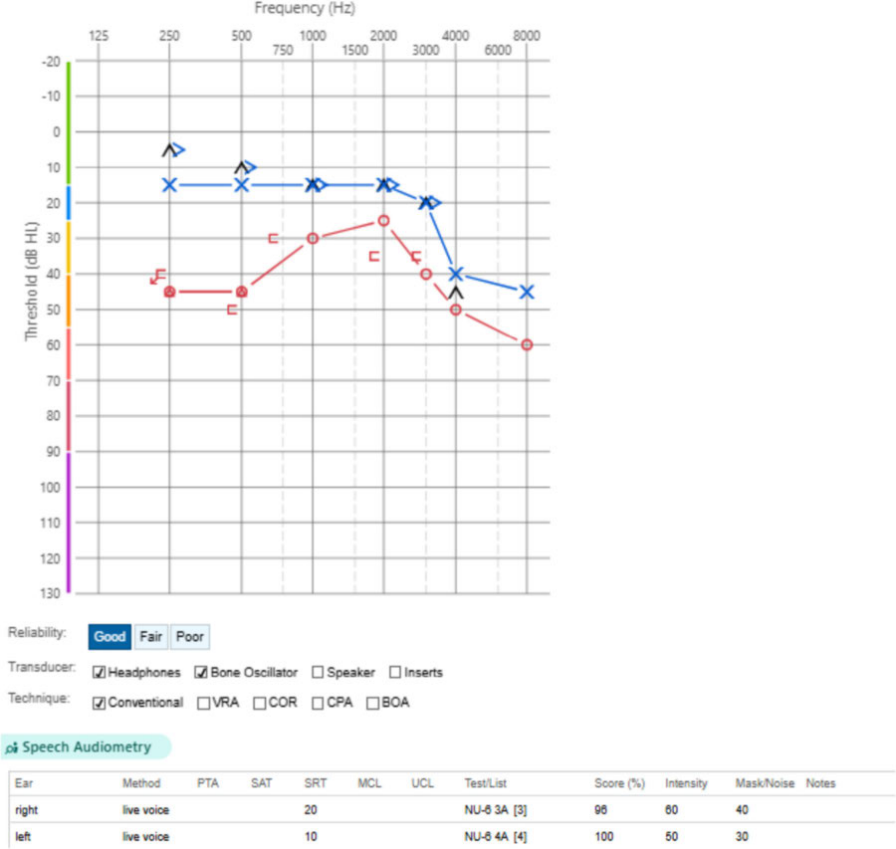
Postoperative pure tone audiogram showing a 5–10 dB midfrequency improvement in hearing.

**Fig. 6:**
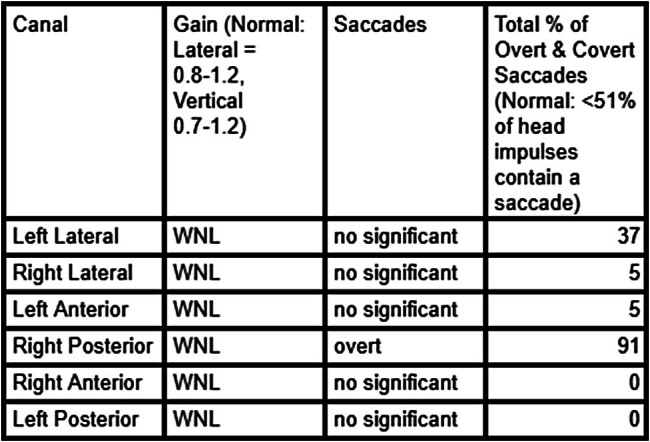
Postoperative vHIT showing improved overt saccades.

While recovering inpatient, she noted subjective improvement in her hearing, which persisted. She had near complete resolution of her balance symptoms, although this took about a month to improve. Postoperative audiogram ( Fig. 5 ) showed a 5- to 10-dB midfrequency improvement in hearing and a normalization of speech discrimination (96%), and vHIT ( Fig. 6 ) showed improved overt saccades in all but the right posterior canal. Interestingly, preoperatively, she had overt saccades in the left lateral and posterior semicircular canals, which also improved postoperatively.

## Discussion

Meniere’s disease is a debilitating form of peripheral vertigo, classically manifesting as recurrent attacks lasting from 20 minutes to several hours, accompanied by sudden, unilateral hearing loss, “roaring” tinnitus, and severe rotational vertigo. While the etiology of Meniere’s disease is not fully elucidated, hydrops is common among these patients. 6 This is a phenomenon in which fluid accumulation within the endolymphatic sac exerts tensile pressure upon the membrane of the sac, into the vestibular aqueduct, and possibly displacing Reissner’s and/or the basilar membrane. 7 While evidence of hydrops is common among those with Meniere’s disease, the severity of symptoms is weakly correlated with the extent of hydrops. Some patients with extensive hydrops experience merely episodic sensorineural hearing loss, and others remain asymptomatic entirely. The variance in symptomology remains unexplained, as does the reason for the fluid accumulation. 8

Meniere’s-like symptoms may also occur from secondary endolymphatic hydrops. Rare case reports associate Meniere’s-like symptoms with petrous temporal masses in the posterior fossa. 8 Meningiomas are common intracranial neoplasms that arise from dural surfaces. About 10% of all meningiomas are located in the posterior fossa and are adherent to the petrous surface of the temporal bone. These lesions may cause a myriad of symptoms due to compression of cranial nerves V–VIII. 8 More anterior-based lesions may cause Tic douloureux, while other lesions may invade the auditory canal and be a mimic of vestibular schwannoma with gradual hearing loss and/or vertigo. More posteriorly located masses tend to be asymptomatic, but sufficiently large lesions can potentially involve the vestibular aperture or endolymphatic sac and may be associated with Meniere’s-like symptoms. 8

Reports of petrous meningiomas leading to Meniere’s symptoms are rare. A search of -indexed literature with the terms “posterior fossa,” “meningioma,” “dizziness,” “vertigo,” and “Meniere’s” from 2005 to 2023 revealed five case series 8 9 10 11 12 and three case reports 13 14 totaling 22 patients with both diagnosed posterior fossa tumors and vertigo ( Table 1 ). Of these cases, 13 describe midline tumors involving the cerebellum, brainstem, or basilar artery, while 11 note petrous tumors involving the endolymphatic sac. Three cases did not specify beyond the presence in the posterior fossa. One retrospective study identified 16 cases of posterior fossa tumors among 2000 presentations of vertigo between 1995 and 2005. Of these patients, tumors were found at the cerebellopontine angle in eleven, cerebellum in two, brainstem in two, and jugular fossa in one. None were explicitly noted to involve the endolymphatic sac. 15

**Table 1:** Summary of prior studies reporting posterior fossa tumors and vertigo.

Study	Design	Patient(s)	Summary
Morshed et al., 2021 8	Retrospective review ( *n* = 7)	Patients with posterior petrous face meningiomas involving the vestibular aperture presented with Meniere’s like symptoms	All patients underwent surgical resection. Six of 7 experienced significant improvement or resolution of vertigo
Konopka et al., 2008 9	Case series ( *n* = 3)	Patients with posterior cranial fossa tumors causing vertigo	All patients underwent audiovestibular tests and were demonstrated to have positional nystagmus indicative of a central lesion
Chang et al., 2009 10	Retrospective review ( *n* = 6)	Patients with posterior fossa lymphoma who presented with vertigo and ataxia	All patients underwent chemotherapy and irradiation. Three patients died, 2 were lost, and 1 was kept alive. Follow-up was not reported
Lee et al., 2014 11	Case series ( *n* = 8)	Patients initially diagnosed with benign paroxysmal positional vertigo were ultimately discovered to have central paroxysmal positional vertigo due to a posterior fossa lesion	Authors sought to bring awareness to posterior fossa lesions as a potential cause of positional horizontal nystagmus in patients refractory to canalith repositioning therapy
Varadarajan et al., 2020 12	Case series ( *n* = 3)	Patients with asymmetric sensorineural hearing loss, episodic vertigo, and tinnitus in the setting of posterior fossa tumors causing endolymphatic hydrops	The authors suggested that electrocochleography could be used intraoperatively to demonstrate endolymphatic hydrops, although immediate improvement may not be detected
Choi et al., 2011 13	Case report ( *n* = 1)	A patient with a posterior petrous meningioma with recurrent vertigo	The patient was treated with transmastoid tumor removal and had resolution of symptoms
Kalani et al., 2018 14	Case report ( *n* = 1)	A patient with headache, vertigo, and nausea was found to have an epidermoid cyst of the posterior fossa	The patient underwent keyhole retrosigmoid craniotomy for tumor removal, but there was no mention of the balance outcome

Posterior fossa tumors can have multiple mechanisms by which they can cause dizziness. Symptoms can be due to an effect on central balance pathways or due to direct effects on the peripheral vestibular system. This patient’s tumor invaded the bone of the vestibular aqueduct but left the dural boundaries of the endolymphatic sac intact.

We postulate that the tumor caused compression of the endolymphatic sac, leading to secondary endolymphatic hydrops. The exact cause of Meniere’s-like symptoms associated with meningiomas overlying the vestibular aqueduct and endolymphatic sac is not well understood, but other authors have suggested possible mechanisms. Morshed et al. 8 suggest direct tumor invasion and hyperostosis of the bone surrounding the endolymphatic system as a cause. Friedman and colleagues suggest that tumor microcirculation may alter endolymphatic homeostasis, leading to symptoms. 16 Another theory is that involvement of the endolymphatic sac may block resorption of endolymph, trigger inflammation in response to intratumoral microhemorrhage, or lead to excess fluid production by the tumor itself. 5

We demonstrate in this case that resection of the tumor can resolve balance-related symptoms and improve hearing. This case report is consistent with prior case reports showing symptomatic improvement after resection, and this case also demonstrates improvement in high-frequency VOR with pre- and postoperative vHIT testing. We are not aware of any prior demonstration of these findings.

## Conclusion

Posterior fossa meningiomas overlying the endolymphatic sac can present with Meniere’s-like symptoms. This case adds to the small body of literature surrounding these tumors. Our case, similar to prior reports, presents a patient who had marked improvement in symptoms after resection as well as objective vestibular function improvement. This supports surgical resection in these unique patients. While our patient presented with a known tumor, MRI imaging to assess for posterior fossa tumors should be considered in patients with Meniere’s-like symptoms unresponsive to medical therapy.
